# Neighborhood factors and use of dental services among young adults in Southern Brazil: a 5-year cohort study

**DOI:** 10.1590/1807-3107bor-2026.vol40.068

**Published:** 2026-08-21

**Authors:** Jocelaine Vieira Vione, Letícia Donato Comim, Nathália Costa de Castro, Giovana Rodrigues Silva, Julio Eduardo do Amaral Zenkner, Luana Severo Alves

**Affiliations:** (a) Universidade Federal de Santa Maria – UFSM, School of Dentistry, Department of Restorative Dentistry, Santa Maria, RS, Brazil.; (b) Universidade Federal de Santa Maria – UFSM, School of Dentistry, Department of Stomatology, Santa Maria, RS, Brazil.

**Keywords:** Oral Health, Health Inequities, Neighborhood Characteristics, Social Determinants of Health, Socioeconomic Factors

## Abstract

This prospective cohort study assessed the influence of contextual variables of the residential neighborhood on the pattern of preventive use of dental services among young adults from southern Brazil. The study was linked to a population-based cross-sectional survey carried out in 2018 that evaluated the oral health conditions of adolescents aged 15–19 years from Santa Maria, southern Brazil (n = 1,197). After a mean (± SD) period of 5 (± 0.5) years, 570 individuals were reassessed (response rate: 47.6%). Questionnaires collected sociodemographic characteristics, dental service use (including the reason for the last visit), and participants’ addresses. The outcome was the last dental visit for prevention, defined as positive responses at both baseline and follow-up. The main predictor variables were contextual neighborhood variables (mean income, percentage of literate inhabitants, percentage of residences with tap water, paved streets, sewerage system, and public lighting). Unadjusted and adjusted multilevel Poisson regression models were used to estimate relative risk (RR) and 95% confidence intervals (CI). Approximately one-quarter of participants reported that their last dental visit was for prevention (n = 151, 26.5%). After controlling for individual-level variables, participants living in neighborhoods with above-median income (RR=1.27; 95%CI: 1.04–1.55; p = 0.02), higher literacy levels (RR = 1.31; 95%CI:1.02–1.68; p = 0.03), greater proportion of paved streets (RR =1.28; 95%CI: 1.01–1.63; p = 0.04), or greater sewerage coverage (RR = 1.40; 95%CI: 1.05–1.85; p = 0.02) were more likely to report their last dental visit for prevention. In conclusion, this cohort study demonstrated that neighborhood-level variables influenced the pattern of preventive dental visits among young adults in southern Brazil.

## Introduction

The context in which individuals live significantly influences their health behaviors, affecting both their perception of the need for care and the adoption of healthy lifestyles.^1^ Structural determinants of health, which include social, economic, and political mechanisms, generate socioeconomic positions and operate through intermediary factors to determine vulnerabilities and exposure to conditions that compromise health.^2^ Population-level analyses based on social determinants of health are essential for public policy, because they guide actions aimed at reducing health inequities.^3^ Similarly, understanding oral health inequities requires a broader perspective that transcends the isolated analysis of individual risk factors, considering the social structures that shape these risk factors.^4^

Previous studies have examined the association between contextual variables and different oral health outcomes in the Brazilian population, including untreated dental caries^5,6^, traumatic dental injuries^7,8^, and oral health-related quality of life^9-11^. However, little is known regarding the association between contextual variables and patterns of dental attendance. A recent systematic review of 11 longitudinal studies showed that regular dental attendance was associated with reduced caries experience, fewer missing teeth, and improved oral health-related quality of life,^12^ which supports the relevance of this outcome. Research conducted in developed countries has indicated an association between favorable neighborhood socioeconomic conditions and the use of dental services,^13-15^ yet data from developing countries such as Brazil are still scarce. To the best of our knowledge, no study has assessed contextual factors at the neighborhood level in relation to the use of preventive dental services in the Brazilian context.

Understanding the transitional phase from adolescence to young adulthood and the challenges it involves is important for planning health interventions that meet the needs of this population. Considering these aspects, the aim of this study was to assess the influence of neighborhood-level contextual variables on the pattern of preventive dental service use among young adults in southern Brazil over a 5-year period. The hypothesis was that young adults residing in neighborhoods with more favorable conditions (higher income, higher literacy levels, and better infrastructure) would be more likely to attend dental visits for preventive care on a regular basis.

## Methods

This study was reported according to the STROBE (Strengthening the Reporting of Observational Studies in Epidemiology) guidelines.^16^

### Study design and sample

This 5-year follow-up cohort study was conducted in Santa Maria, a mid-sized city in southern Brazil. The baseline was in 2018, with adolescents aged 15–19 years attending public and private schools. The sample size calculation considered a prevalence of 50% ("worst case scenario"), 95% confidence interval (CI), 80% power, and 3% precision level. It was estimated that 1,066 students would be required, to which a non-participation rate of 50% was added, resulting in 1,600 adolescents to be invited. All 37 urban schools were invited, of which 31 agreed to participate (22 public and 9 private).

Adolescents born between 1999 and 2003, enrolled in the regular school year, and attending any school period (morning, afternoon, or night) were considered eligible. The number of participants per school was proportional to the number of enrolled students. Individuals with fixed orthodontic appliances or with special needs were not considered eligible. A list of all eligible schoolchildren was compiled for each school, and a random sample was selected using a random number generator (www.random.org). In total, 1,197 adolescents were included in the cross-sectional study.

### Follow-up assessment

At the 5-year follow-up, all baseline participants were considered eligible. Several search strategies were adopted to achieve the largest possible number of follow-up participants. Initially, contact was attempted through phone calls, email, and social networks (Facebook, Instagram, and WhatsApp). As a final strategy, home visits were conducted at the addresses recorded in the questionnaires.

Once contact was established, individuals were invited to participate in this second phase of the study. Those who agreed were examined either at the School of Dentistry of the Federal University of Santa Maria or at their residence, depending on their availability and preference. Considering a 95% CI, 80% power, an expected proportion of unexposed individuals with a 20% prevalence of outcome, and an expected risk estimate of 2.0, the minimum sample size for this follow-up was 374 individuals.

### Data collection

Data were collected at baseline (March–November 2018) and follow-up (December 2022–April 2024), with a mean (± SD) interval of 5 ± 0.5 years. At both time points, participants self-completed a structured questionnaire addressing sociodemographic characteristics and dental service use. Demographic characteristics included sex (male or female), age (≤ 16 years or ≥ 17 years, dichotomized by the median/mean), and skin color, based on Brazilian criteria,^17^ later dichotomized as White or others (Black, Brown, Asian, or Indigenous). Socioeconomic data included mother's education (≤ 8 years or > 8 years of schooling) and family socioeconomic status (SES), measured by the Brazilian Economic Classification Criterion. Households were classified as low (≤ 16 points, social class DE), mid-low (≥ 17 to ≤ 22 points, class C2), mid-high (≥ 23 to ≤ 28 points, class C1), or high (≥ 29 points, class A, B1, and B2).^18^ SES was dichotomized as low/mid-low or mid-high/high for analysis. Regarding dental service use, participants were asked to state the reason for their last dental visit, with the following options: check-up/prevention, toothache, extraction, fillings, or other. This questionnaire also collected their residential address to define the neighborhood according to official sources.^19^ The questionnaire was previously tested in a similar population and adjusted for comprehension.

Clinical examinations to record the decayed, missing, or filled teeth (DMFT) index^20^ were performed at both time points. Participants were examined in a supine position, after tooth cleaning and drying, under artificial light, using a millimeter probe. Two examiners conducted the baseline examinations (minimal kappa 0.80 for intra- and inter-examiner reliability), and two others (L.D.C. and N.C.C.), trained and calibrated by one of the baseline examiners, conducted the follow-up examinations (minimum kappa 0.83 for inter-examiner and intra-examiner reliability). Training was based on photographs and clinical examinations, and calibration was verified through duplicate examinations in adolescents/young adults not included in the study sample, with a 7-day interval between exams.

### Neighborhood contextual data

Santa Maria covers 1,780.194 sq. km., with an estimated population of 271,735 in 2022.^21^ The urban area comprises 42 neighborhoods distributed across 8 administrative regions. At baseline, official data were collected for each neighborhood: mean income, percentage of literate inhabitants (functional literacy based on the question "Can you read and write?"), percentage of residences with tap water, percentage of paved streets, percentage of residences with a sewerage system, and percentage of streets with public lighting^17^.

### Data analysis

Analyses were conducted in STATA (Stata 14.2, StataCorp, College Station, USA). A weight variable based on the probability of selection and population distribution by sex and SES was used to adjust for potential bias in population estimates.^22^ Differences between followed participants and those lost to follow-up were assessed with the chi‐squared test and further examined with bootstrap sensitivity analysis.

The study outcome was the last dental visit for prevention. The question ‘What was the reason for your last visit to the dentist?’ was used to define this outcome. Participants who answered ‘check-up/prevention’ at both time points (baseline and follow-up) were classified as ‘yes,’ whereas those who selected this option only once or not at all were classified as ‘no.’ This approach distinguished consistent preventive attendance from chance responses.

The main predictors were the neighborhood contextual variables described above. Continuous variables (mean income, literacy rate, tap water, paved streets, sewerage, and public lighting) were dichotomized at the median, classifying neighborhoods as ≤ median (unfavorable) or > median (favorable). Individual-level predictors (demographic and socioeconomic) were considered adjusting variables. All predictors were collected at baseline.

Multilevel Poisson regression analysis was used to assess the influence of contextual neighborhood variables on preventive attendance, considering adolescents as first-level units and city regions as second-level units. The multilevel model applied fixed effects with a random intercept. Unadjusted and adjusted relative risks (RR) and 95% CIs were estimated. One adjusted model was run for each contextual variable because of collinearity. Variables with *p* ≤ 0.20 in the unadjusted analysis were included in the adjusted model.^23^ SES was retained in the adjusted models because mother's education and SES were highly correlated, with SES showing a lower p-value in the unadjusted analysis.

### Ethical aspects

The study protocol was approved by the Research Ethics Committee of the Federal University of Santa Maria (CAAE 43938021.0.0000.5346). All participants signed informed consent, received a report of their oral health status, and were referred for treatment when needed.

## Results

Of the 1,197 adolescents assessed at baseline, 570 were reevaluated at the 5-year follow-up (cohort retention rate: 47.6%). The main reasons for loss to follow-up were refusal to participate (n = 303), inability to contact participants (n = 216), and relocation to another city (n = 108) (Figure). Baseline characteristics of participants lost to follow-up and those retained are compared in Table 1. Statistically significant differences were observed for age, skin color, maternal education, and SES. Retained participants were significantly younger, White, had mothers with higher levels of education, and had higher SES than those lost to follow-up. Sensitivity analysis using bootstrap simulation indicated that these differences did not influence the study results. The retained individuals had a mean (± SD) DMFT of 1.23 (± 1.90) at baseline and 1.91 (± 2.66) at follow-up. Median DMFT values (interquartile range) were 0 (0, 0) at baseline and 1 (0, 3) at follow-up.

**Figure:**
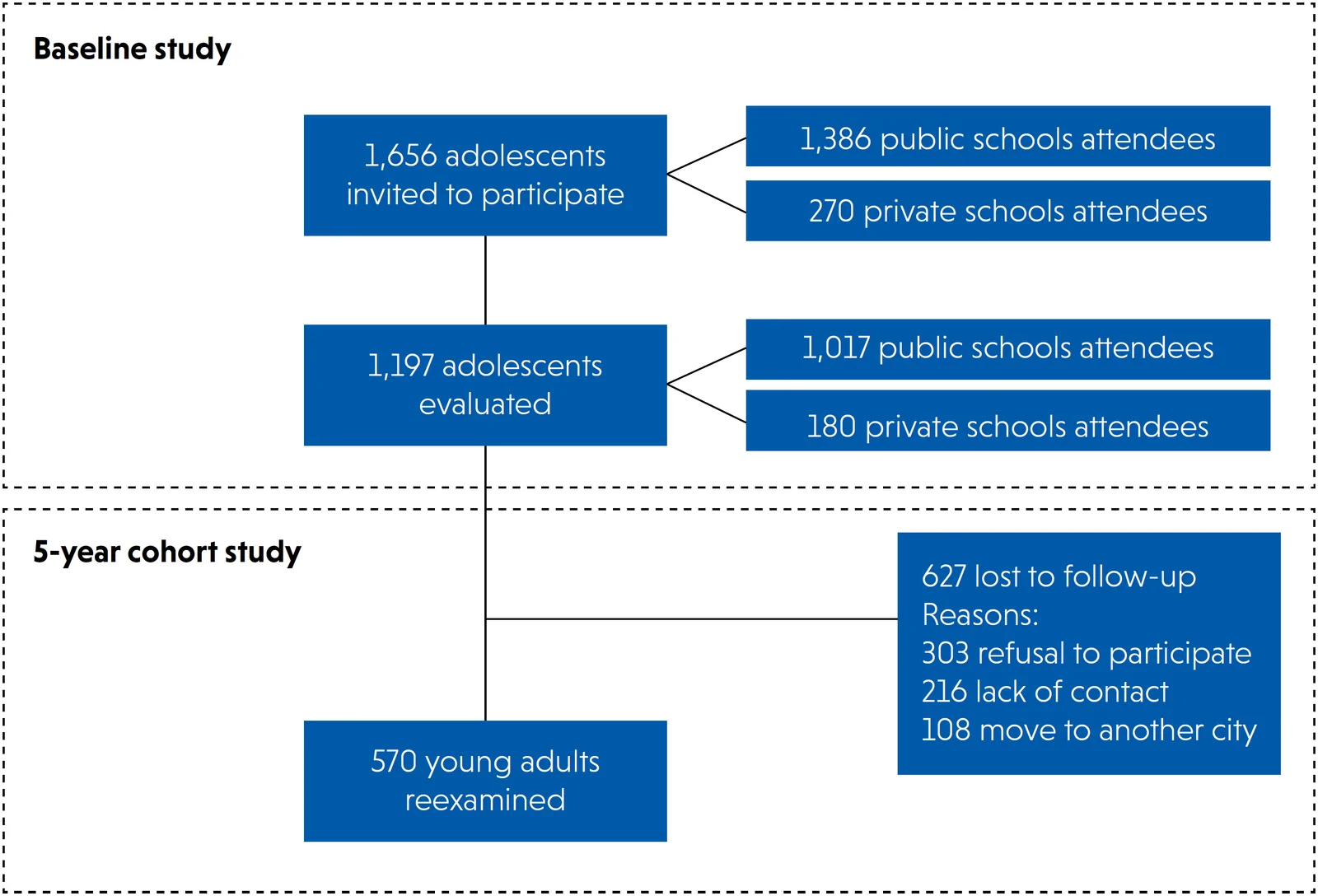
Study flowchart.

**Table 1:** Comparison of baseline sociodemographic characteristics of followed individuals and those lost to follow-up.

Variables		Followed		Lost to follow-up		p-value**
		(n = 570)		(n = 627)		
		n	%	n	%	
Sex						
	Male	237	41.6	276	44.0	
	Female	333	58.4	351	56.0	0.39
Age (years)						
	≤ 16	331	58.1	324	51.7	
	≥ 17	239	41.9	303	48.3	0.03
Skin color*						
	White	405	72.8	374	61.6	
	Others	151	27.2	233	38.4	< 0.001
Maternal education (years)*						
	≤ 8	241	44.0	336	55.9	
	> 8	307	56.0	295	44.1	< 0.001
Socioeconomic status						
	Low/mid-low	214	37.5	307	49.0	
	Mid-high/high	356	62.5	320	51.0	< 0.001

* Missing data;

** Chi-squared test.

The distribution of contextual- and individual-level characteristics and the unadjusted multilevel Poisson regression analysis are presented in Table 2. Among the 570 participants, 26.5% (n = 151) reported that their last dental visit was for preventive care at both baseline and follow-up. This proportion was significantly higher among participants living in neighborhoods with a greater percentage of paved streets and a greater percentage of residences with sewerage systems.

**Table 2:** Sample distribution, the percentage of participants who last visited the dentist for prevention by predictor variables and the unadjusted multilevel Poisson regression analysis.

Variables			n (%)	Last visit to the dentist for check-up/prevention (%)	RR (95%CI)	p-value
Contextual-level variables (Neighborhood)						
Mean income*						
		≤ median	275 (50.5)	63 (22.9)a	1.00	
		> median	269 (49.5)	81 (30.1)a	1.38 (1.06–1.80)	0.02
% Literate inhabitants*						
		≤ median	250 (46.0)	57 (22.8)a	1.00	
		> median	294 (54.0)	87 (29.6)a	1.40 (0.99–2.00)	0.06
% Tap water						
		≤ median	280 (50.9)	73 (26.1)a	1.00	
		> median	270 (49.9)	72 (27.7)a	0.99 (0.62–1.58)	0.98
% Paved streets*						
		≤ median	261 (48.0)	59 (22.6)a	1.00	
		> median	283 (52.0)	85 (30.0)b	1.32 (0.98–1.77)	0.07
% Sewerage system*						
		≤ median	277 (50.9)	60 (21.7)a	1.00	
		> median	267 (49.1)	84 (31.5)b	1.39 (0.95–2.04)	0.09
% Public illumination						
		≤ median	265 (50.2)	74 (27.9)a	1.00	
		> median	263 (49.8)	65 (24.7)a	0.89 (0.74–1.07)	0.21
Individual-level variables						
	Sex					
		Male	237 (41.6)	70 (29.5)a	1.00	
		Female	333 (58.4)	81 (24.3)a	0.94 (0.67–1.32)	0.72
	Age (years)					
		≤ 16	331 (58.1)	103 (31.1)a	1.00	
		≥ 17	239 (41.9)	48 (20.1)b	0.62 (0.45–0.88)	0.006
	Skin color*					
		White	151 (27.2)	36 (23.8)a	1.00	
		Others	405 (72.8)	114 (28.2)a	1.13 (0.95–1.34)	0.17
	Maternal education (years)*					
		≤ 8	241 (44.0)	54 (22.4)a	1.00	
		> 8	307 (56.0)	93 (30.3)b	1.35 (1.03–1.77)	0.03
	Socioeconomic status					
		Low/mid-low	214 (37.5)	42 (19.6)a	1.00	
		Mid-high/high	356 (62.5)	109 (30.6)b	1.63 (1.35–1.98)	<0.001
	Total		570 (100)	151 (26.5)		

RR: relative risk; CI: Confidence interval.

* Missing data. Different letters indicate statistically significant difference between categories (chi-square test, p ≥ 0.05).

At the contextual level, unadjusted multilevel Poisson regression models showed that individuals residing in neighborhoods with above-median income were 38% more likely to report a last dental visit for prevention than those from neighborhoods with below-median income (RR = 1.38; 95% CI: 1.06–1.80; p = 0.02). At the individual level, younger participants (≤ 16 years), those whose mothers had more than 8 years of education, and those with higher SES were more likely to report that their last dental visit was for prevention.

Table 3 presents the results of the adjusted multilevel Poisson regression analyses. After adjusting for age, skin color, and SES, most contextual neighborhood variables were significantly associated with the study outcome. Participants living in neighborhoods with above-median income (RR = 1.27; 95%CI: 1.04–1.55; p = 0.02), higher literacy levels (RR = 1.31; 95%CI: 1.02–1.68; p = 0.03), a greater proportion of paved streets (RR = 1.28; 95%CI: 1.01–1.63; p = 0.04), or greater sewerage coverage (RR = 1.40; 95%CI: 1.05–1.85; p = 0.02) were more likely to report that their last dental visit was for prevention. Access to tap water and public lighting at the neighborhood level did not influence preventive dental service use in this population.

**Table 3:** Adjusted multilevel Poisson regression analysis investigating the influence of neighborhood variables on the last visit to the dentist for prevention.

Variables		RR (95%CI)*	p-value
Mean income			
	≤ median	1.00	
	> median	1.27 (1.04–1.55)	0.02
% Literate inhabitants			
	≤ median	1.00	
	> median	1.31 (1.02–1.68)	0.03
% Paved streets			
	≤ median	1.00	
	> median	1.28 (1.01–1.63)	0.04
% Residences with sewerage system			
	≤ median	1.00	
	> median	1.40 (1.05–1.85)	0.02

RR: relative risk; CI: Confidence interval.

* Estimates are adjusted for age, skin color, and socioeconomic status.

## Discussion

This prospective cohort study assessed whether contextual factors of the residential neighborhood influenced the use of preventive dental services among young adults from southern Brazil. The results showed that individuals residing in neighborhoods with higher mean income, higher literacy levels, and better infrastructure (a greater proportion of households with paved streets and sewerage coverage) were more likely to report that their last dental visit was for preventive care compared with those living in neighborhoods with opposite characteristics, thereby supporting the study hypothesis. To the best of our knowledge, this is the first study in a developing country to evaluate contextual neighborhood factors in relation to the use of preventive dental services among this age group while also addressing neighborhood structural characteristics.

The transition from adolescence to adulthood has significant implications for health, both during this period and throughout life.^24^ This process reflects the biological and social transformations of adolescence and is influenced by social determinants as well as by risk and protective factors that shape health-related habits in adulthood.^25^ This stage consolidates behavioral and lifestyle patterns and involves continuous processes of cognitive and social development, including changes in family dynamics, personal relationships, education, and professional life.^24,26^ Despite the relevance of this period, epidemiological surveys assessing this age group are scarce. In Brazil, national oral health surveys (1986, SB Brazil 2003, 2010, and 2023) monitored adolescents aged 15–19 years and adults aged 35–44 years.^27^ This approach creates a gap in knowledge regarding the oral health of young adults, highlighting the importance of studies focused on this population in the Brazilian context.

Our findings showed that young adults living in neighborhoods with higher mean income and higher literacy levels were more likely to report their last dental visit for preventive care, underscoring the relevance of socioeconomic context in shaping oral health behaviors. This result is consistent with previous studies conducted in developed countries, indicating that residents of neighborhoods with better socioeconomic conditions tend to have greater adherence to preventive care practices and better oral health outcomes.^13-15^ A study conducted in the United States (USA) analyzed the association between neighborhood poverty and adolescent use of dental care using multilevel modeling in two large national datasets. Even after adjustment for individual factors, such as family income, health insurance, and parental education, adolescents living in poor neighborhoods were less likely to use dental care than those living in non-poor neighborhoods.^13^ Another USA study based on national data investigated the association between neighborhood-level factors and dental visits in young adults.^14^ After adjustment for individual-level factors, neighborhood education level was significantly associated with dental service use in the random-effects model. In the Netherlands, a cohort study showed that living in disadvantaged neighborhoods was associated with a significantly increased risk of dental caries and a tendency toward fewer dental visits.^15^

It has been suggested that residents of neighborhoods with lower SES may be more exposed to adverse conditions throughout life, including structural and social barriers that limit access to essential resources such as transportation, healthy food, and social support, which in turn negatively affect quality of life and oral health perception.^28-31^ Importantly, neighborhood variables such as mean income and literacy level influenced the use of preventive dental services among young adults in southern Brazil even after adjustment for individual SES. This highlights the contextual influence of place of residence on oral health outcomes, irrespective of individual conditions.

Neighborhood infrastructure also played a significant role in the pattern of dental visits observed in this study. Greater coverage of paved streets and sewerage systems in neighborhoods, which indicate better housing conditions, was positively associated with the outcome. O’Sullivan et al. suggested that better housing resources in the urban environment contribute to the promotion of population well-being.^32^ Conversely, the absence of such infrastructure reflects limited accessibility and inadequate sanitation, reinforcing social inequities.^33^ Adequate health standards require the implementation of policies and administrative measures aimed at improving neighborhood conditions, since better housing can prevent disease, enhance quality of life, and support the achievement of the Sustainable Development Goals (SDGs), particularly those related to health (SDG 3) and sustainable cities (SDG 11).^34,35^ Housing has increasingly been recognized as a determinant of health and is now considered a key element in the implementation of intersectoral public health programs and primary prevention.^35^ A cross-sectional population-based study conducted by our research group showed that adolescents from southern Brazil living in residences without paved streets or tap water were more likely to perceive poorer oral health-related quality of life.^11^ However, that study relied on self-reported individual-level data, whereas the present research expands the analysis to neighborhood-level contextual factors. No influence of tap water or public lighting was observed here.

This study presents some limitations. First, the attrition rate and the significant differences between participants retained and those lost to follow-up must be acknowledged. Despite extensive efforts to contact participants, fewer than 50% of the original sample was reassessed. Tracking participants is a major challenge in cohort studies and becomes even more critical in age groups no longer linked to schools. In addition, the transition from adolescence to young adulthood is a period of major transformation, with substantial behavioral and social changes, new personal bonds, career definition, entry into the job market, family formation, and parenthood. These aspects may explain why this age group is traditionally underrepresented in the dental literature. Although the retained participants tended to have more favorable sociodemographic profiles, sensitivity analysis showed that these differences did not influence the study results. Even so, it can be speculated that the proportion of young adults visiting the dentist for preventive purposes would likely have been lower if a greater number of vulnerable individuals had been retained. This potential bias was minimized using a weighting variable derived from the probability of selection and population distribution by sex and SES.

Although sociodemographic variables were included in the adjusted models, residual confounding cannot be excluded. Important individual-level factors such as participant education, employment status, or access to dental insurance were not assessed and may have influenced the observed associations. Another consideration is that the outcome relied on self-reports and was therefore strongly dependent on participants’ recall of the reason for their last dental visit. Recall bias or social desirability bias could not be ruled out. However, because the questionnaires were self-completed without interviewer interference, these biases may have been reduced. Finally, it could be argued that most contextual variables showed no association with the study outcome until adjustment for individual factors, which challenges the hierarchy of determinants. Nevertheless, this finding aligns with the theoretical framework guiding our study. According to the Commission on Social Determinants of Health (CSDH) Conceptual Framework^2^, structural determinants, including contextual variables, operate through intermediary determinants at the individual level to shape health outcomes. Therefore, it is expected that the direct effect of neighborhood-level contextual factors on preventive dental visits would emerge only after accounting for individual-level mediators such as socioeconomic position and access to resources.

The study also presents strengths. Its prospective cohort design and the specific age group under investigation stand out. Furthermore, its pioneering evaluation of structural neighborhood variables represents an important contribution. Another strength lies in the definition of the outcome by combining baseline and follow-up data, which helped identify individuals consistently seeking preventive dental care. Although this approach diverges somewhat from the conventional cohort logic—in which baseline represents exposure and follow-up represents outcome—it allowed us to isolate the subgroup regularly attending preventive visits.

From a practical perspective, these findings reinforce the importance of public policies that extend beyond individual-focused interventions to address contextual conditions. Improving neighborhood infrastructure and strengthening educational initiatives may create environments more conducive to the adoption of preventive behaviors. Future research should further investigate the mechanisms through which contextual factors influence oral health behaviors and the pathways mediating these associations.

## Conclusion

In conclusion, this 5-year cohort study demonstrated that contextual neighborhood factors influenced the use of preventive dental services among young adults in southern Brazil. Individuals living in neighborhoods with higher mean income, higher literacy levels, and better infrastructure were more likely to attend preventive dental visits.

## Data Availability

The datasets generated during and/or analyzed during the current study are available from the corresponding author on reasonable request.
